# Characteristics of IL-9 induced by *Schistosoma japonicum* infection in C57BL/6 mouse liver

**DOI:** 10.1038/s41598-017-02422-8

**Published:** 2017-05-24

**Authors:** Lu Li, Hongyan Xie, Mei Wang, Jiale Qu, Hefei Cha, Quan Yang, Yuanfa Feng, Yanwei Qi, Huaina Qiu, Nuo Dong, Jun Huang

**Affiliations:** 1 Guangdong Provincial Key Laboratory of Allergy & Clinical Immunology, Sino-French Hoffmann Institute, The Second Affiliated Hospital, Guangzhou Medical University, 511436 Guangzhou, China; 2 Functional Experiment Centre, Guangzhou Medical University, 511436 Guangzhou, China; 3 Affiliated Xiamen Eye Center & Eye Institute of Xiamen University, 361001 Xiamen, China; 4 Key Laboratory of Protein Modification and Degradation, School of Basic Medical Sciences, Guangzhou Medical University, Guangzhou, 511436 China

## Abstract

Liver granulomatous inflammation and fibrosis were the primary pathological changes observed during *Schistosoma japonicum (S. japonicum)* infection. In the present study, the characteristics of IL-9 were investigated in the liver of *S. japonicum* infection C57BL/6 mice. Immunofluorescence, qRT-PCR, and ELISA results demonstrated that the expression of IL-9 significantly increased after infection (*P* < 0.01). FACS results indicated that the peak of IL-9^+^ Th9 cells in the liver mononuclear cells appeared at the early phase of infection (week 5), except that Th9 cells, CD8^+^ Tc cells, NKT and γδT cells could secrete IL-9 in this model. Although IL-9 neutralization has a limited effect on liver granulomatous inflammation, it could decrease the level of fibrosis-associated factor, PC-III, in the serum of infected mice (*P* < 0.05). Taken together, our results indicated that IL-9 was an important type of cytokine involved in the progression of *S. japonicum* infection-induced hepatic damage.

## Introduction

Schistosomiasis japonica is a chronic helminth infection of humans caused by *S. japonicum*. Although this disease has been controlled in many countries, it remains common in China, where over 50 million people are currently infected, as well as the Philippines and small pockets of Indonesia^1–3^. The primary pathological changes during *S. japonicum* infection are granulomatous inflammation and progressive fibrosis in liver. Additionally, the latter is a key reason for the induction of serious complications and death. The pathology of this disease is induced by CD4^+^ Th2 cell-driven response, with IL-4 and IL-13, as the dominant cytokines responsible, whereas IL-10, IFN-γ, IL-13Rα2 and regulatory T cells act to limit schistosome-induced pathology^4, 5^. In recent studies, it was reported that increased Th17 cell populations were correlated with more severe pathology and IL-17 neutralization in an *S. Japonicum* infected mouse model could decrease granulomatous inflammation^6–8^.

IL-9 was discovered more than 20 years ago and initially described as a T cell and mast cell growth factor, produced by activated Th2 clones *in vitro* and during Th2-like T cell responses *in vivo* ^9^. TGF-β interactions with IL-1 family members (IL-1α, IL-1β, IL-18, and IL-33) could trigger IL-4-independent IL-9 production by mouse CD4^+^ T cells^10^. IL-4 signalling promoted Th9 cell differentiation, in part, by suppressing the ability of TGF-β to induce the expression of the T regulatory (TReg) cell-associated transcription factor fork head box P3 (FOXP3)^11^. Recently, IL-9 was thought to be pleiotropic in function, and it can stimulate mast cell accumulation in tissues, promote ILC survival, enhance class switching to IgE in B cells, and alter haematopoietic progenitor cell activity, as its receptor is expressed on multiple cell types^12^. Except Th9 cells, IL-9 could be produced by mast cells, T_C_9′ cells, natural killer T (NKT) cells, and other CD4^+^ T cells, including Th17 cells and Foxp3^+^ T regulatory (Treg) cells^12^.

As a prototypical type 2 cytokine, IL-9 has been implicated in many diseases, especially helminth infections. In individuals with lymphatic filariasis, antigen-specific Th9 cells were detected^13^. Licona-Limon *et al*. showed that Th9 cells are able to cause rapid *Nippostrongylus brasiliensis* worm expulsion in Rag2-deficient hosts, indicating a critical and non-redundant role for Th9 and IL-9 during parasitic worm infection^14^. In IL-9-deficient mice, after inducting pulmonary granuloma formation around the Schistosoma eggs, when compared with controls, the expression of Th1 and Th2 cytokines remained unchanged^15^. During murine *schistosome mansoni* infection, IL-9 expression leads to Th2 cytokine-dominated responses, which leads to death^16^.

However, studies on the relationship between Th9/IL-9 and *S. Japonicum* infection are scarce: there are limited data to show the dynamics of Th9 cells after *S. japonicum* infection. In this study, we observed the characteristics of IL-9 induced by *Schistosoma japonicum* infection in the liver of C57BL/6 mice.

## Materials and Methods

### Mice, parasites and infection

Six- to eight-week-old female C57BL/6 mice were purchased from Laboratory Animal Centre of Sun Yat-sen University (Guangzhou, China) and maintained in a specific pathogen-free microenvironment at the Laboratory Animal Centre, Guangzhou Medical University. Sixty mice were percutaneously infected with 40 ± 5 *Schistosoma japonicum* cercariae that were obtained from *Oncomelania hupensis-*infected snails (purchased from Jiangsu Institute of Parasitic Disease, Wuxi, China). All infected mice were administered intragastric injection with praziquantel (360 mg/kg·day) for three days from day 31 after infection. Another six infected mice were divided into an infected/anti-IL-9 mAb-treated group. Anti-IL-9-treated mice received the first intraperitoneal injection of 30 µg anti-IL-9 mAb (Biolegend, San Diego, CA, USA) 5 weeks after *S. japonicum* infection, then, the same dose every three days, for a total of 6 times. Twenty pathogen-free mice were used as a control group. All protocols for animal use were approved to be appropriate and humane by the institutional animal care and use committee of Guangzhou Medical University (2011-44). Animal experiments were performed in strict accordance with the regulations for the Administration of Affairs Concerning Experimental Animals, and all efforts were made to minimize suffering.

### Antibodies

APC-cy7 conjugated anti-mouse CD3 (145-2C11), PerCP-Cy5.5 conjugated anti-mouse CD4 (RM4-5), APC conjugated anti-mouse CD4(565994), APC conjugated anti-mouse CD8 (555369), FITC conjugated anti-mouse γδTCR (553177), PE-cy7 conjugated anti-mouse NK1.1 (PK136), APC conjugated anti-mouse IFN-γ (XMG1.2), APC conjugated anti-mouse IL-9 (d9302c12), PE conjugated anti-mouse IL-9(561463), and isotype-matched control monoclonal antibodies (X39, G155-178) were purchased from BD Pharmingen (San Diego, CA, USA).

### Isolation of hepatic lymphocytes

Mice were respectively sacrificed on week 4, 5, 6, 7, 8 and 9 after infection, and single hepatic lymphocyte suspensions were made for flow cytometry analysis. The precava was cut and then sterile saline was injected to remove blood from the livers. Livers were mechanically dissociated and processed through a 100 μm cell strainer (BD Falcon). Hepatic lymphocytes were isolated by lymphocyte separation medium. Isolated cells were washed twice in Hank’s balanced salt solution and diluted to a final concentration of 2 × 10^6^ cells/ml in complete RPMI-1640 medium, which contained 10% heat-inactivated foetal calf serum, 100 U/ml penicillin and 100 μg/ml streptomycin.

### RNA preparation for RT-PCR

RNA was extracted from the hepatic lymphocytes of infected and normal mice with Trizol Reagent (Invitrogen Life Technologies, Carlsbad, CA, USA) following the manufacturer’s instructions. The cDNA was synthesized with a SuperScript III Reverse Transcriptase Kit (Qiagen, Valencia, CA). The primers were synthesized from Invitrogen (Shanghai, China) as follows: for IL-9, 5-CTCTCCGTCCCAACTGATGA-3 (forward) and 5-GGTCTGGTTGCATGGCTTTT-3 (reverse); for β-actin, 5-CCGTAAAGACCTCTATGCCAAC-3 (forward) and 5-GGGTGTAAAACGCAGCTCAGTA-3 (reverse). Amplification was performed using the CFX96 touch q-PCR system (Bio-Rad, Hercules, CA, USA) under the following conditions: (1) 1 min at 95 °C;(2) 5 s at 95 °C; (3) 30 s at 60 °C with plate read; (4) 40 cycles for (2) and (3); (5) Melt Curve 65 to 95 °C, increment 0.5 °C for 5 s with plate read. Q-PCR products were analysed on a 1.0% multi-welled agarose gel. Electrophoresis was carried out in 1 × TAE buffer at 400 V for 30 min. The gel was visualized in the ChemiDoc XRS Universal Hood II (Bio-Rad Laboratories).

### Detection of cell surface markers and intracellular cytokines

Single hepatic cell suspensions were incubated for 5 h in the presence of phorbol 12-myristate 13-acetate (PMA) (20 ng/ml, Sigma), ionomycin (1 μg/ml, Sigma) and brefeldin A (10 μg/ml, Sigma) at 37 °C under a 5% CO_2_ atmosphere. Next, cells were washed twice in PBS and stained with PerCP-cy5.5-anti-CD4 mAb, APC-cy7-anti-CD3 mAb, APC-anti-CD8 mAb, PE-cy7-anti-NK1.1 mAb and FITC-anti-γδT mAb for 30 min at 4 °C in the dark. The next step, cells were fixed with 4% paraformaldehyde, and permeabilized overnight at 4 °C in PBS buffer containing 0.1% saponin (Sigma), 1% BSA, and 0.05% NaN_3_. The next day, cells were stained with conjugated antibodies specific for the cytokines IFN-γ and IL-9. The stained hepatic lymphocytes were analysed using flow cytometry (FACS; BD Aria II) and data were analysed by the FlowJo version 7.6 software (Tree Star, USA).

### Histochemistry

The experimental procedures used for routine histological examination of liver tissue were previously described^7^. Briefly, livers were removed and later fixed in 10% formalin, embedded in paraffin, and sectioned. The sections were then examined by light microscopy under 100 × or 200 × magnification after haematoxylin and eosin staining. The size of single hepatic egg granulomas was measured by computer-assisted morphometric analysis using DP-2BSW software (Olympus, Tokyo, Japan). Eighty to 100 granulomas were measured under a microscope for each group and only a visible central egg was analysed for accuracy. Granuloma size is expressed as the mean area ± SD.

For immunofluorescence detection of the Th9 cells in hepatic granuloma, fixed livers were embedded in optimal cutting temperature compound (OCT, Sakura Finetec Japan, Tokyo, Japan), frozen in liquid nitrogen and then sliced and stored at -70 °C before use. When ready for labelling, the sections of liver tissue were air-dried at room temperature for 1 h, roasted over flame 3 times, and then air-dried for another 10 min. Sections were then treated with blocking buffer (10% foetal calf serum in PBS) at room temperature for 1 h. After being washed with PBS three times, the sections were incubated with APC conjugated anti-mouse CD4 and PE conjugated anti-mouse IL-9 (BD, Inc., San Diego, CA) in a 1:50 dilution overnight in the dark at 4 °C. The next step, sections were washed with PBS three times. Then, the sections were treated with DAPI (10 µg/ml) for 10 min. Finally, the sections were washed with PBS three times and mounted with 50% glycerol/PBS. Fluorescent staining patterns were detected and acquired by confocal laser scanning microscope, CLSM (Leica, TCS SP8 X).

### Cytokine measurement by Bio-Plex Multiplex System

Serum samples from mice infected at different points-in-time were collected to analyse the Th1/Th2 cytokines’ profile using the Bio-Plex suspension array system (Bio-Rad Laboratories Inc., Hercules, CA, USA). The Bio-Plex Assay Kit, purchased from Bio-Rad, was applied to detect the Th1/Th2 cytokines including IL-2, IL-4, IL-5, IL-10, IL-12, IFN-γ, TNF-α, and GM-CSF in serum samples of infected mice and uninfected controls. The measurement method was performed according to the manufacturer’s instructions. Briefly, a 12.5 µl sample was diluted 1:4 with sample diluent, incubated with antibody-coupled beads and washed. Next, the samples were incubated with biotinylated secondary antibodies, followed by incubation with streptavidin phycoerythrin. The beads readings were analysed using the Luminex System (Bio-plex 200, Bio-Rad), and the data were analysed by the Bio-Plex Manager Software (Bio-Rad).

### ELISA detection

Levels of IL-9 in the supernatant of cultured cells were detected by ELISA according to the manufacturer’s instructions (88-8092-22, eBioscience). The procollagen-III (PC-III) and hyaluronic acid (HA) in serum were analysed by ELISA according to the manufacturer’s instructions (202124, Usbiological, abx052387 abbexa). Samples were read at 450 nm using a microplate reader (Moder ELX-800, BioTek).

### Statistics

Data were analysed using SPSS (v11.0). Statistical evaluation of the difference between means was performed by unpaired, two-tailed Student’s *t*-tests; P < 0.05 was considered to be significant.

## Results

### Dynamic changes of granulomatous inflammation during *S. japonicum* infection

**Figure 1:**
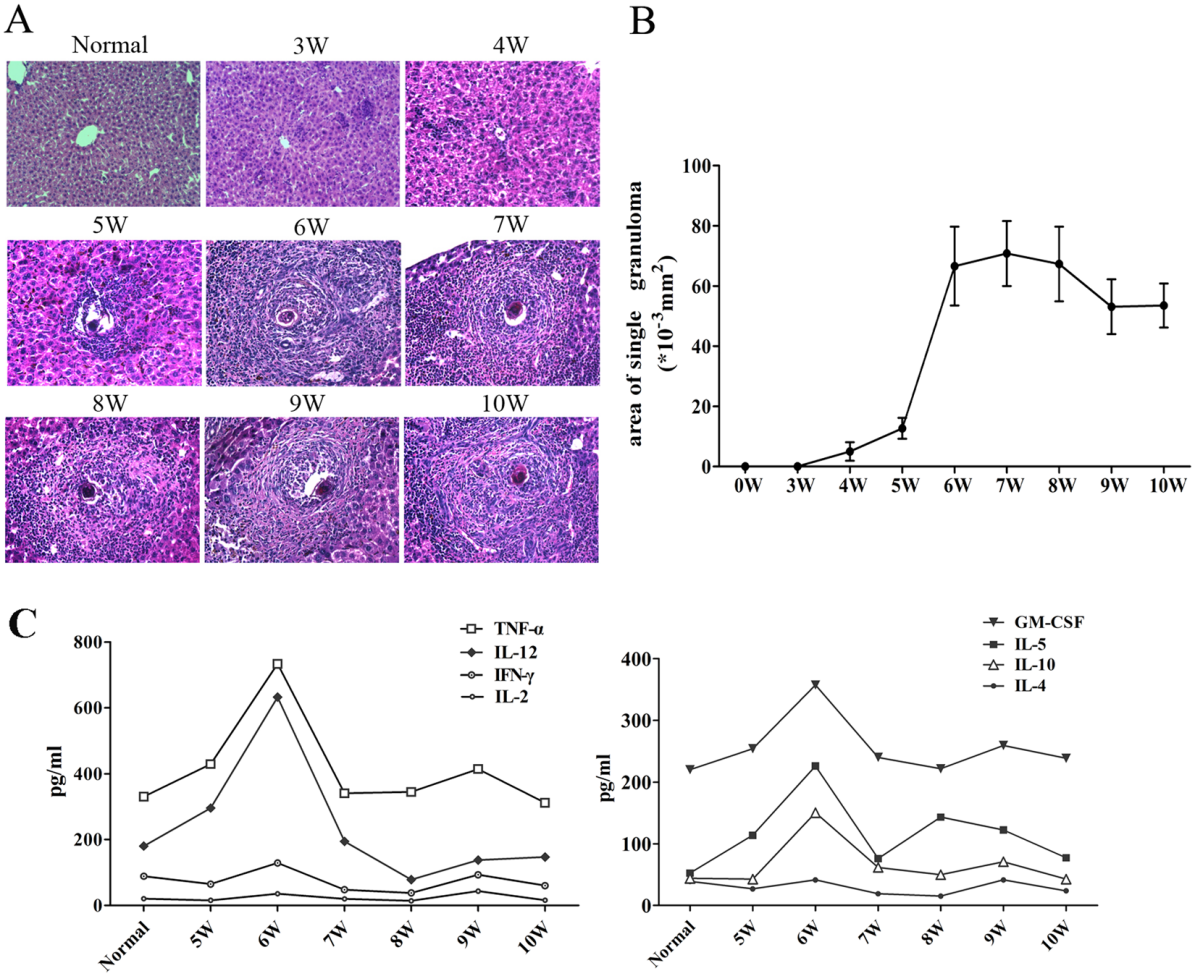
Dynamic changes of granulomatous inflammation during *S. japonicum* infection. Female C57BL/6 mice were infected with 40 ± 5 *S*. *japonicum* cercariae per mouse. These mice were sacrificed at 3, 4, 5, 6, 7, 8, 9, and 10 weeks post infection, and uninfected mice were used as control. (**A**) Mice livers were fixed in paraformaldehyde, embedded in paraffin, and then sliced and examined by haematoxylin and eosin staining. Representative photomicrographs are shown at x200 magnification. (**B**) Sizes of single egg granuloma were measured by computer-assisted morphological analysis. Only granulomas with a visible central egg were analysed for accuracy. Between 80 and 100 granulomas for each group were measured under a microscope. The results are expressed as mean ± SD (n = 5). (**C**) The levels of Th1/Th2 cytokines in mice serum were determined by using Bio-Plex multiplex system; *P < 0.05. Three independent experiments were performed, and representative results are shown.

*S. japonicum* infection can result in hepatic granulomatous inflammation and fibrosis. To investigate the relationship between granulomatous inflammation and infection period, the dynamic changes of the single egg granuloma area in mice livers were analysed. Haematoxylin and eosin (HE) staining of liver sections revealed the normal cellular organization of uninfected hepatic lobules, with the typical actinomorphic distribution of hepatic cord centred around central veins. In infected livers, a few small and mixed inflammatory cell foci appeared on week 3, and Schistosome eggs without granulomatous reaction could be observed on week 4. On week 5, egg granulomas appeared (mean cross-sectional area: 12.7 ± 3.5 × 10^−3^ mm^2^). With the extension of the infection period, more and larger egg granulomas and many more inflammatory cells gradually appeared in mice livers (Fig. 1A). The single egg granuloma area reached a maximum on weeks 6, seven and 8 (week 6, 66.6 ± 13.1 × 10^−3^ mm^2^; week 7, 70.8 ± 10.8 × 10^−3^ mm^2^; week 8, 67.3 ± 12.4 × 10^−3^ mm^2^). However, on week 9, the area began to decrease (week 9, 53.1 ± 9.1 × 10^−3^ mm^2^; week 10, 53.5 ± 7.3 × 10^−3^ mm^2^, Fig. 1B). At the same time, serum from each group of mice was collected and levels of Th1/Th2 cytokines were detected by Bio-Plex Multiplex System, as described in Materials and Methods. The results (Fig. 1C) showed that compared to others the changes of TNF-, Il-12, IL-5, and GM-CFS were significant, and the level of these cytokines in serum increased on week 5 after infection, peaked on week 6, and then began to decrease. The serum level of IL-5 had a significant rebound on week 8.

### Detection of IL-9 in *S. japonicum*-infected mouse liver

**Figure 2:**
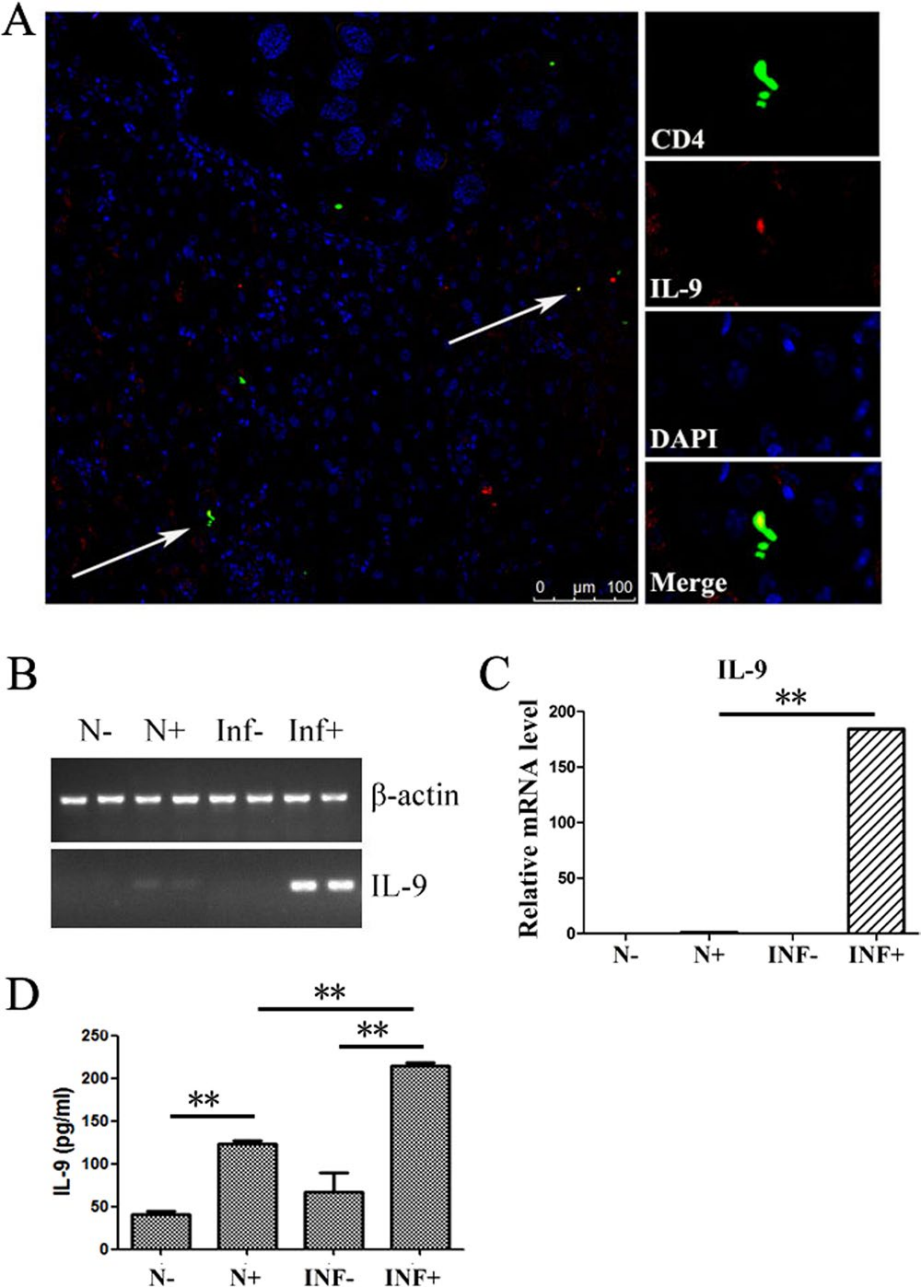
Detection of Th9 cells and IL-9 in livers of *S. japonicum-* infected mice. Female C57BL/6 mice were infected with 40 ± 5*S. japonicum* cercariae per mouse. These mice were sacrificed at 5-6 weeks post infection. (**A**) A representative result of immunofluorescence. Livers were embedded in O.C.T, and then sliced and examined by fluorescent antibody staining. CD4 and IL-9 are identified by green fluorescence (APC) and red fluorescence (PE), respectively, whereas nuclei are visualized using DAPI staining (blue). CD4^+^IL-9^+^ cells are found around granulomas (white arrows). (**B**/**C**) The expression of IL-9 genes in mice hepatic lymphocytes was detected by qRT-PCR. The hepatic lymphocytes from normal (N) and 5–6 weeks-infected mice (INF) were prepared and incubated for 5 hours with PMA and ionomycin (+) or not (−), respectively. The data are representative of three independent experiments. (**D**) Levels of IL-9 in supernatant of cultured hepatic lymphocytes. The hepatic lymphocytes from normal (N) and 5–6 weeks-infected mice (INF) were prepared and stimulated by anti-CD3 plus anti-CD28 for 72 hours (+) or not (−), respectively. Concentration of IL-9 in the supernatants of cultured cells was detected by ELISA. One representative result of 3 to 5 independent results are shown; **P < 0.01.

To detect the existence of IL-9 in *S. japonicum*-infected mouse liver, C57BL/6 mice were sacrificed at 5–6 weeks after *S. japonicum* infection. Livers were picked out, and ice-slices were made. PE-labelled anti-mouse IL-9, APC-labelled anti-mouse CD4, and DAPI were used to do immunofluorescence. Results showed that some IL-9^+^ cells (red) and, seldom, some IL-9^+^ CD4^+^ Th cells (yellow) resided around Schistosome eggs (Fig. 2A). Moreover, hepatic lymphocytes from normal and 5-6 weeks-infected mice were prepared and incubated for 5 hours with PMA and ionomycin or not, respectively. RNA was collected, cDNA were transcribed, and the level of IL-9 mRNA was detected by qRT-PCR. As shown in Fig. 2B, the IL-9 gene stripe of stimulated, infected hepatic lymphocytes was brighter than others. The qRT-PCR results indicated that the expression of IL-9 mRNA significantly increased after *S. japonicum* infection (Fig. 2C). Additionally, hepatic lymphocytes from normal and infected mice were isolated and cultured with or without stimulation of CD3 plus CD28 for 72 h. The cell supernatants were collected and the level of IL-9 was detected by ELISA. As shown in Fig. 2D, the concentration of IL-9 of stimulated hepatic lymphocytes was 123.395 ± 3.765 pg/ml (normal mice), and increased to 215 ± 2.89 pg/ml (infected mice, *P* < 0.01).

### Kinetic analysis of hepatic Th9 cells during *S. japonicum* infection

**Figure 3:**
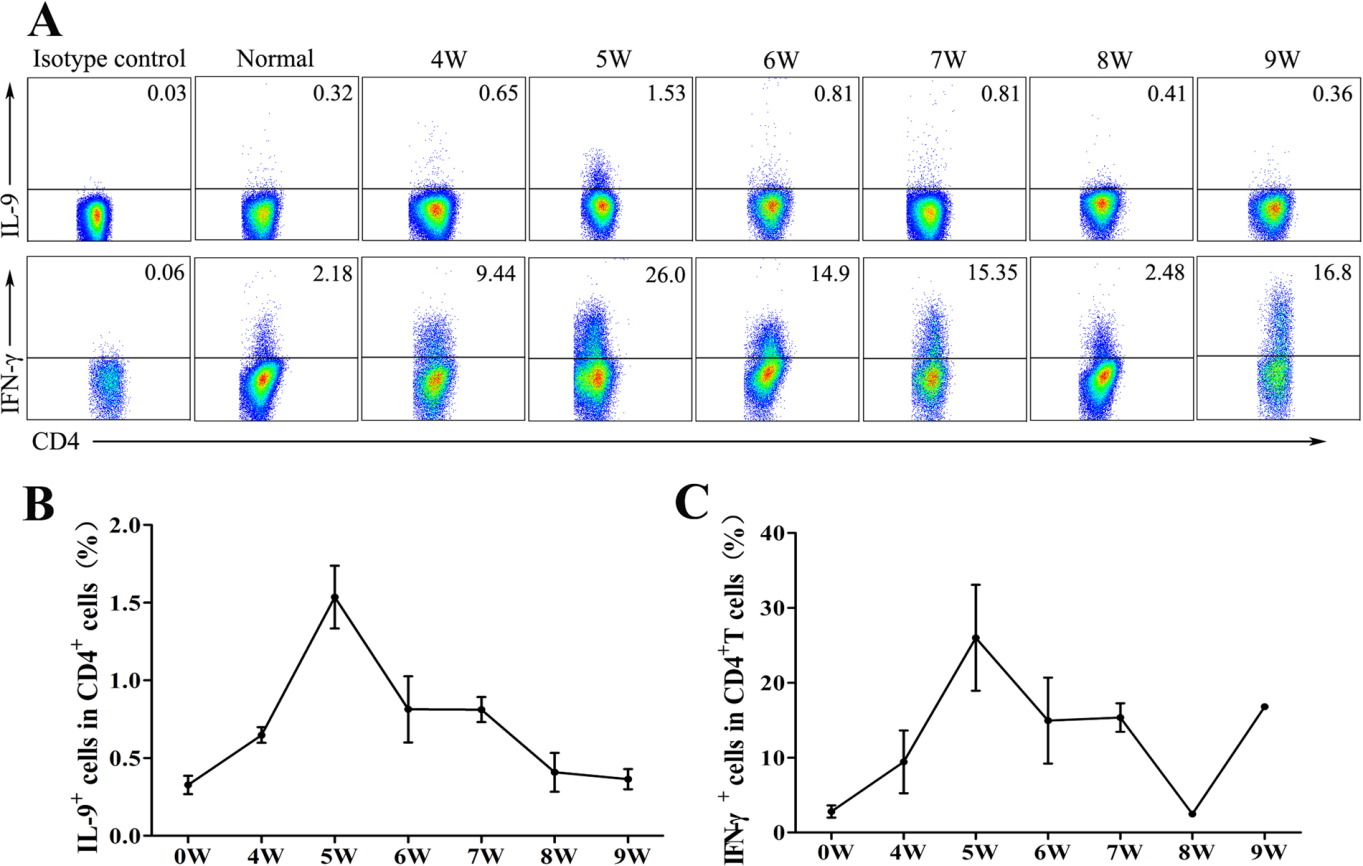
Dynamic changes of Th9 and Th1 cells in hepatic lymphocytes during *S. japonicum* infection. Female C57BL/6 mice were infected with 40 ± 5*S. japonicum* cercariae per mouse. These mice were sacrificed at 4, 5, 6, 7, 8 and 9 weeks post-infection and uninfected mice were used as control. Single hepatic lymphocyte suspensions were stimulated with PMA and ionomycin supplemented with BFA for 5 hours. Th9 cells were determined by using flow cytometry. (**A**) A representative flow plots and gating strategy are shown. (**B**,**C**) The kinetics of the percentages of Th1 and Th9 cells from three independent experiments with similar results. Data represent the mean ± SD. One representative result of three independent results are shown.

To observe the changes in Th9 cells during *S. japonicum* infection, lymphocytes were isolated from blood-free livers at 4, 5, 6, 7, 8 and 9 weeks after infection (normal control was set), and stimulated with PMA plus ionomycin for 5 hours, and intracellular cytokines staining (ICS) was done. Cells were gated on CD3^+^ CD4^+^ cells first, and the expression of IL-9 and IFN-γ was detected. The results (Fig. 3) showed that the percentage of Th9 cells increased on week 4 (0.65 ± 0.04%), reached the maximum on week 5 (1.53 ± 0.20%), decreased on week 6 to 0.81 ± 0.21%, which was close to the value on week 7 (0.81 ± 0.08%), and then continued to decrease to 0.41 ± 0.13% and 0.36 ± 0.07% on week 8 and week 9, respectively. Changes of IFN-γ^+^ Th1 cells were similar to Th9 cells.

### Source of IL-9 in *S. japonicum*-infected mouse liver

**Figure 4:**
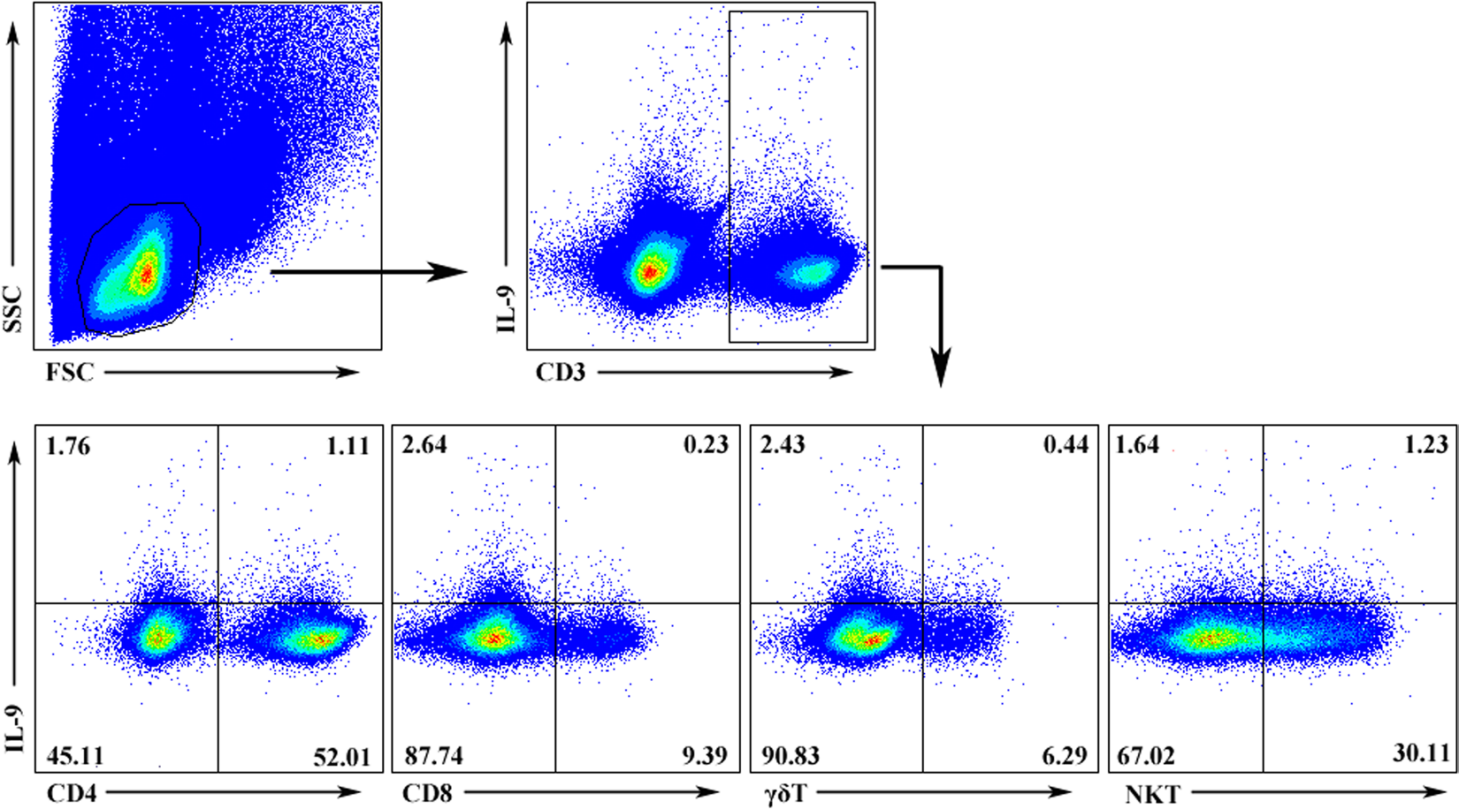
IL-9 expression of different T cell subsets in infected hepatic lymphocytes. Female C57BL/6 mice were infected with 40 ± 5 *S. japonicum* cercariae per mouse. These mice were sacrificed at 5–6 weeks post infection. Single hepatic lymphocyte suspensions were stimulated for 5 hours with PMA and ionomycin supplemented with BFA. Cells were stained with different fluorophore-conjugated antibodies for CD3, CD4, CD8, γδTCR, NK1.1 and IL-9 for FACS analysis. Numbers in quadrants are percentages of cells in each expression phenotype. A representative of three independent experiments is shown.

To detect the source of IL-9 in *S. japonicum*-infected mouse liver, single hepatic lymphocyte suspensions of infected mice were stimulated with PMA and ionomycin. Next, cells were stained with different fluorescent- conjugated antibodies for FACS analysis. First, CD3^+^ T cells were gated. Then, the proportion of IL-9^+^ cells in CD4-, CD8-, NK1.1-, and γδTCR-positive cells was detected, respectively. As shown in Fig. 4, CD4^+^ Th cells, CD8^+^ Tc cells, NKT and γδT cells could secrete IL-9 after non-specific stimulation during *S. japonicum* infection. The percentage of IL-9^+^CD4^+^ T cells detected by FACS analysis was 1.11%, which was close to the proportion of IL-9^+^ NKT cells (1.23%), and higher than IL-9^+^ CD8^+^ Tc cells (0.23%) and γδT cells (0.07%).

### Roles of IL-9 in *S. japonicum* infection-induced hepatic granulomatous and fibrosing inflammation

**Figure 5:**
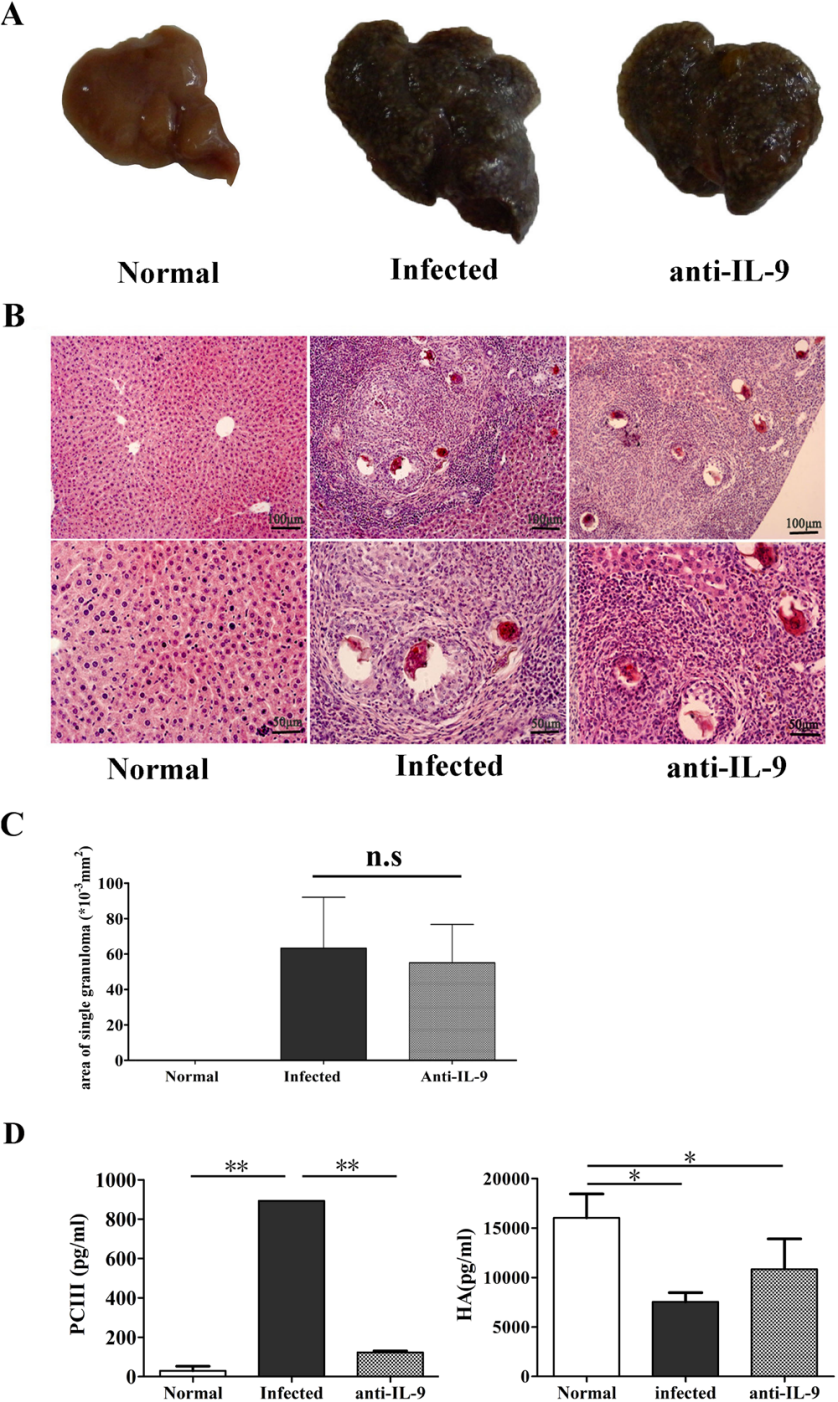
Roles of anti-IL-9 in *S. japonicum* infection-induced hepatic granulomatous and fibrosing inflammation. Female C57BL/6 mice were randomly divided into three groups: normal group, infected group and anti-IL-9 group. The infected group and anti-IL-9 group were infected with 40 ± 5 *S. japonicum* cercariae per mouse. From week 5 post-infection, the control IgG mAb or anti-IL-9 mAb were injected intraperitoneally into the infected group and anti-IL-9 group mice every 3 days, for a total of 6 times, respectively. These mice were sacrificed at 8 weeks post-infection. Mice livers were fixed in paraformaldehyde, embedded in paraffin, and then sliced and examined by haematoxylin and eosin staining. (**A**) The gross appearance of livers from the three groups. (**B**) Representative photomicrographs are shown at x100 magnification for upper panels and ×200 for lower panels. (**C**) Sizes of single egg granuloma were measured by computer-assisted morphometric analysis. Only granulomas with a visible central egg were analysed for accuracy. The results are expressed as mean ± SD (6 mice per group); n.s, *P* > 0.05. (**D**) Levels of pro-collagen type III (PC-III, left) and hyaluronic acid (HA, right) in the serum of mice were detected by ELISA. The results are expressed as mean ± SD; *P < 0.05, **P < 0.01. One representative result of 3 to 5 independent results is shown.

To explore the role of IL-9 in hepatic granulomatous inflammation, we divided mice into three groups: control, infected and infected/anti-IL-9 mAb-treated mice. These mice were sacrificed 8 weeks after infection and livers were isolated. First, we compared the gross appearance of livers from different groups (Fig. 5A). Normal liver was light red with smooth surface. In contrast, the infected liver was dark red with many small white spots on its surface, indicating that infected mice were experiencing severe inflammation and egg pyogenic granulomas were formed in their liver. Additionally, livers from infected anti-IL-9 mAb-treated mice seemed a little shallower than the infected ones, but there were still many white spots on the surface (Fig. 5A). The sections of livers were detected by haematoxylin and eosin staining (Fig. 5B). Normal livers, showed the regular actinomorphic distribution of hepatic cord, centring around central veins. However, there were many Schistosome eggs in livers from infected mice and infected/anti-IL-9 mAb-treated mice. Around these eggs, there were a variety of inflammatory cells, which formed the granulomatous inflammation. We compared the size of a single egg granuloma in mice livers (Fig. 5C) and found that the mean size of a single granuloma from infected/anti-IL-9 mAb-treated livers was 55.04 ± 18.59 × 10^−3^ mm^2^, slightly smaller than the infected group (63.34 ± 20.65 × 10^−3^ mm^2^). However, there was no significant difference between the two groups.

The level of PC-III and HA were detected by ELISA (Fig. 5D). Serum of control, infected and infected/anti-IL-9 mAb-treated mice were collected on week 8 post infection. The level of PC-III in infected mice was significantly higher than control and anti-IL-9 groups (infected: 893.06 ± 23.42 pg/ml; Normal: 29.64 ± 19.42 pg/ml; anti-IL-9: 122.71 ± 8.25 pg/ml, Fig. 5D). In contrast, HA level in normal control mice was significantly higher than in infected mice and anti-IL-9-treated mice (Normal: 16044.5 ± 2413.21 pg/ml; infected: 7540.27 ± 930.73 pg/ml; anti-IL-9: 10840.8 ± 3064.02 pg/ml, Fig. 5D). HA level in anti-IL-9-treated mice was higher than the infected mice, however, there was no significant difference between these two groups. Hence, the results indicate that IL-9 might influence the hepatic fibrosis by promoting the synthesis and accumulation of PC-III, whereas it moderately regulates HA expression during *S. japonicum* infection.

## Discussion

Liver granuloma response is considered to be a specific mark of Schistosome infection^17^, and this inflammatory process is the key pathological change of the disease. Most of the time, severity and progression of disease are determined by the size of granuloma and its component. In our model of *S. japonicum* infection, we used praziquantel intragastric injection to establish the status of chronic *S. japonicum* infection in mice. As shown in Fig. 1A/B, the size of a single egg granuloma increased slowly from three weeks post-infection to five weeks post-infection, but then increased very rapidly between five and six weeks post-infection and remained at peak between six and eight weeks post-infection. These results indicated that the granulomatous inflammation is extraordinarily active during this period, as reported in many previous studies, and further implied that 6 weeks after *S. japonicum* infection is a good time point to observe the induced granulomatous inflammation.

It has been reported that both type 1 cytokines, such as IL-2, IL-12, IFN-γ, and type 2 cytokines, such as IL-4, IL-5, IL-10, IL-13, are involved in the liver egg granulomas in *S. Mansoni* infection^18–20^. TNF-α is a kind of multifunctional cytokine secreted by microphage. The excessive secretion of TNF-α can induce the hyperplasia of granulocytes and fibroblasts, injure endothelial cells and promote the generation of IL-1, leading to shock, tissue injury, fibrosis, etc. In *S. japonicum*-infected BALB/c mice, IL-12 is considered to play an impeditive role in granuloma formation and fibrosis^21^. Therefore, we detected the levels of Th1/Th2 cytokines in serum during this process. The results showed that the expressions of TNF-α, IL-12, IL-5, IL-10 and GM-CSF in serum increased from five weeks post-infection and to peak levels at week 6 post-infection; however, as typical type 1 cytokines, the levels of IL-2 and IFN-γ in serum did not change during the key period. The similar situation appears with IL-4, a typical type 2 cytokine (Fig. 1C). These results are consistent with the granulomatous formation results (Fig. 1A/B), which demonstrated that week 6 is a good time point to observe *S. japonicum* infection-induced inflammatory response in mouse liver. The small change of typical Th1/2 cytokines in mouse serum is related to the low level of these cytokines in serum, as detected in previous clinical research^22, 23^, and also suggested that these typical Th1/2 cytokines primarily affect the site of local inflammation.

As mentioned before, IL-9 was a prototypical type 2 cytokine, which has been implicated in many diseases, especially helminth infections. The existence of IL-9 in *S. japonicum*-infected mouse liver was detected in this study. Immunofluorescent results (Fig. 2A), PCR results (Fig. 2B/C), and ELISA results (Fig. 2D) indicated that *S. japonicum* infection could induce IL-9 in mouse liver, as detected in *Schistosoma mansoni-*infected mice^16^. Moreover, the percentage of IL-9-secreted CD4^+^ Th9 cells was kinetically observed in *S. japonicum-* infected mouse liver by ICS. As shown in Fig. 3, the percentage of Th9 cells increased on week 4 and reached the maximum on week 5. The time of IL-9^+^ Th9 cells’ peak in infected mouse liver was one week before the peak of the granuloma area in liver and cytokine level in serum and implied that IL-9 could mediate the pathogenic changes induced by *S. japonicum*.

Additionally, it was reported that IL-9 could be produced by mast cells, T_C_9′ cells, natural killer T (NKT) cells, and other CD4^+^ T cells, including Th17 cells and Foxp3^+^ T regulatory (Treg) cells^12^. Even innate lymphoid cells are a significant source of IL-9^24^. In this study, the expression of IL-9 was compared in CD4^+^ T cells, CD8^+^ T cells, NKT cells and γδT cells in r *S. Japonicum-*infected C57BL/6 mice liver. As shown in Fig. 4, the percentage of IL-9^+^CD4^+^ T cells was close to the proportion of IL-9^+^ NKT cells (1.23%) and higher than IL-9^+^ CD8^+^ Tc cells (0.23%) and γδT cells (0.07%). A similar study, showed that peripheral iNKT cells can adopt IL-9 producing NKT cell phenotype, which was able to mediate pro-inflammatory effects *in vivo* ^25^, and suggested that NKT cell was a main source of IL-9 in *S. Japonicum-*infected C57BL/6 mice liver. For NKT cells, it is a kind of innate lymphocyte, implying that NKT cells might be producing IL-9 sooner than Th9 cells in response to invading *S. Japonicum*. Although, it was reported that IL-9^+^ γδT cells increased in asthmatic mice, demonstrating that γδT cells may promote inflammation by secreting IL-9^26^. The percentage of IL-9^+^ γδT cells in infected mouse liver is 0.44% in CD3^+^ T cells, which means that γδT cells are not a main source of IL-9 in *S. Japonicum-*infected mouse liver. However, this figure is approximately 7% in γδT cells, which is higher than other cell populations (*P* < 0.05). It is suggested that γδT cells are apt to secrete IL-9, and might play some function in *S. Japonicum-*infected induced liver damage.

Granuloma formation is the result of a host adaptive immune response mediated by CD4^+^ T cells specific for Schistosome egg Ags (SEA), which damaged hepatocytes and destroyed the normal histological structure of the liver. Although livers from infected anti-IL-9 mAb-treated mice appeared slightly shallower than the infected ones (Fig. 5A), no obvious difference in the size of a single egg granuloma in mice liver was detected between the two (*P* > 0.05), and these results differ from our findings in IL-17 blockage mice^7^; which, indicated that the effect of IL-9 to induce inflammation in *S. Japonicum-*infected mouse liver is limited, and may be related to the powerful function of IL-7 in recruiting inflammatory cells^27^. The lower percentage of IL-9^+^ cells (Fig. 3) and lower concentration of IL-9 induced (Fig. 2D) in the infected mice liver lymphocytes may be another reason. Additionally, hyaluronic acid (HA), type III pre-collagen (PC III), collagen IV (C IV) and laminin (LN), as serum markers, were widely used in the diagnosis of liver fibrosis in patients with chronic viral infections or alcoholic liver diseases^28^. Results (Fig. 5D) demonstrated that IL-9 antibody could obviously reduce the level of PC-III in mouse serum (*P* < 0.05), and suggested that IL-9 could influence hepatic fibrosis by promoting the synthesis and accumulation of PC-III in the course of *S. japonicum* infection. Similarly, aberrant expression of cytokine interleukin 9 was found along with interleukin 4 and interferon γ in connective tissue disease-associated interstitial lung disease (association with severity of pulmonary fibrosis)^29^. Serum levels of IL-9 cytokine showed no significant differences (*p* > 0.05) between chronic clinical forms of *Schistosoma mansoni* infection disease, and in comparisons with the different classifications of periportal fibrosis^30^.

## Conclusions

We demonstrated that the IL-9^+^ lymphocyte population significantly expands in the liver of C57BL/6 mice during infection by *S. japonicum*. IL-9-producing cells might mediate the immune response induced by *S. japonicum* in mouse liver. Anti-IL-9 mAb therapy may be helpful in preventing inflammation-mediated liver damage caused by schistosomiasis.
